# Ice-associated norovirus outbreak predominantly caused by GII.17 in Taiwan, 2015

**DOI:** 10.1186/s12889-017-4869-4

**Published:** 2017-11-07

**Authors:** Hao-Yuan Cheng, Min-Nan Hung, Wan-Chin Chen, Yi-Chun Lo, Ying-Shih Su, Hsin-Yi Wei, Meng-Yu Chen, Yen-Chang Tuan, Hui-Chen Lin, Hsu-Yang Lin, Tsung-Yen Liu, Yu-Ying Wang, Fang-Tzy Wu

**Affiliations:** 1 Taiwan Centers for Disease Control, No.6, Linsen S. Rd., Jhongjheng District, Taipei, Taiwan; 2 Taiwan Food and Drug Administration, No.161-2, Kunyang St, Nangang District, Taipei, Taiwan; 3 Research and Diagnostic Center, Taiwan Centers for Disease Control, No.161, Kunyang St., Nangang Dist, Taipei City, 115 Taiwan

**Keywords:** Norovirus, GII.17, Ice, Water borne, Outbreak, Gastroenteritis

## Abstract

**Background:**

On 5 March 2015, Taiwan Centers for Disease Control was notified of more than 200 students with gastroenteritis at a senior high school during excursion to Kenting. We conducted an outbreak investigation to identify the causative agent and possible vehicle of the pathogen.

**Methods:**

We conducted a retrospective cohort study by using a structured questionnaire to interview all students for consumed food items during their stay at the resort. Students were defined as a gastroenteritis case while having vomiting or diarrhea after the breakfast on 4 March. We inspected the environment to identify possible contamination route. We collected stool or vomitus samples from ill students, food handlers and environmental specimens for bacterial culture for common enteropathogens, reverse transcription polymerase chain reaction (RT-PCR) for norovirus and enzyme-linked immunosorbent assay (ELISA) for rotavirus. Norovirus PCR-positive products were then sequenced and genotyped.

**Results:**

Of 267 students enrolled, 144 (54%) met our case definition. Regression analysis revealed elevated risk associated with iced tea, which was made from tea powder mixed with hot water and self-made ice (risk ratio 1.54, 95% confidence interval 1.22–1.98). Ice used for beverages, water before and after water filter of the ice machine and 16 stool and vomitus samples from ill students were tested positive for norovirus; Multiple genotypes were identified including GI.2, GI.4 and GII.17. GII.17 was the predominant genotype and phylogenetic analyses showed that noroviruses identified in ice, water and human samples were clustered into the same genotypes. Environmental investigation revealed the ice was made by inadequate-filtered and un-boiled water.

**Conclusions:**

We identified the ice made by norovirus-contaminated un-boiled water caused the outbreak and the predominant genotype was GII.17. Adequately filtered or boiled water should be strongly recommended for making ice to avoid possible contamination.

## Background

Norovirus is the leading global cause of acute diarrheal disease as well as gastroenteritis outbreaks [1, 2]. Previously, the most common genotype in norovirus outbreaks worldwide and in Taiwan was GII.4, although new variants emerge every 2–4 years [3, 4]. The GII.17 genotype has historically been an infrequent cause of sporadic norovirus cases, but the emergence of a new GII.17 variant was recognized in several east Asian countries, including China, Japan, Korea, and Taiwan since 2014 [5, 6] and speculated as potentially causing the next norovirus epidemic [7, 8]. In Taiwan, norovirus GII.17 has been identified in one major gastroenteritis outbreak in February 2015 [9].

On 5 March 2015, Taiwan Centers for Disease Control (Taiwan CDC) was notified of more than 200 patients of acute gastroenteritis among students of a senior high school who stayed and ate breakfast at a resort on 4 March and 5 March during a graduation trip in Kenting, a famous tourist attraction in southern Taiwan. Norovirus was suspected because of the predominance of vomiting in clinical presentations. Because another student group from a different school staying and dining at the same resort also reported cluster of gastroenteritis simultaneously on 5 March, we conducted an outbreak investigation to identify the source and possible vehicle of the pathogen.

## Methods

### Epidemiological investigation

We conducted a retrospective cohort study by using a structured questionnaire on 6 March to interview all students who participated in the field trip and surveyed all students on their consumed food items on 4 and 5 March breakfasts, as well as demographics, time of breakfast, symptoms and time of their illness onset.

Students were defined as cases if they had vomiting or diarrhea (having three or more loose or liquid stools per day) and the symptoms started after 4 March breakfast. Bivariate analysis was done by using logistic regression analysis with a log-binominal model to identify suspicious food items. Multiple logistic regression analysis including the variables with *p* value <0.20 in bivariate analysis was then performed to identify associations between suspicious food items and illness. Data were analyzed by using Stata 12.0 (Stata Corp, College Station, TX).

### Environmental inspections

The environmental inspection was performed into two stages: Firstly, the local health department conducted inspection on 6 March after the outbreak notification. They examined the environment of the resort restaurant by checking the requirement of Good Hygienic Practices [10], and collected environmental samples, including swabs of food preparation surfaces, utensils and water samples.

After identifying suspicious food items based on epidemiological investigation, we organized a second environmental inspection on 10 March to review processes of food preparation and water sources to identify possible sources and route of contamination.

### Laboratory examination

Stool specimens from ill students and food handlers were submitted to Taiwan CDC for bacterial and viral tests. Vomitus specimens were also collected and sent for viral tests. Bacterial examinations included cultures for common enteropathogens, such as *Salmonella*, *Shigella*, *Vibrio cholera*, *Vibrio parahemolyticus*, pathogenic *E. coli*, and *Bacillus cereus* [11] while viral tests included reverse transcription polymerase chain reaction (RT-PCR) for norovirus and enzyme-linked immunosorbent assay (ELISA) for rotavirus as previously described [11, 12]. Noroviruses were identified by primer pairs G1SKF/R and G2SKF/R for the detection of norovirus GI and GII, respectively [13].

To determine the identity of the virus strains found in this outbreak with those detected during the same period globally, norovirus positive specimens were selected for full-length ORF2 analysis. RT-PCR was performed using G2SKF-Clone and TX30SXN primers as describe previously [12]. Nucleotide sequences of complete ORF2 were aligned with Clustal W using Kimura’s two parameters. Phylogenetic trees were generated from full-length ORF2 nucleotide sequences using the neighbor joining method by MEGA 4.0 software, and bootstrap values were computed with 1000 replications [14]. The genotypes were determined by submitting the sequences and comparing with references either by RIVM (the Dutch National Institute for Public Health and the Environment) Norovirus genotyping tool or GenBank. The GenBank accession numbers of the GII.17 sequences from patient 1~4, 6~13, 16~19 are MF996723-MF996738 and the GI strains sequences from patient 5, 10, 11, 14, 15 are MF996717- MF996722.

Environmental samples, including samples from cooking utensils and water supply systems in the restaurant and resort, were submitted to Taiwan Food and Drug Administration (Taiwan FDA) for the same bacterial culture and viral tests for norovirus, hepatitis A virus, astrovirus, and sapovirus [15, 16]. For those norovirus G1SKF/R and G2SKF/R PCR-positive specimens, their PCR products were then sequenced and genotyped with the same methods as Taiwan CDC.

## Results

**Fig. 1:**
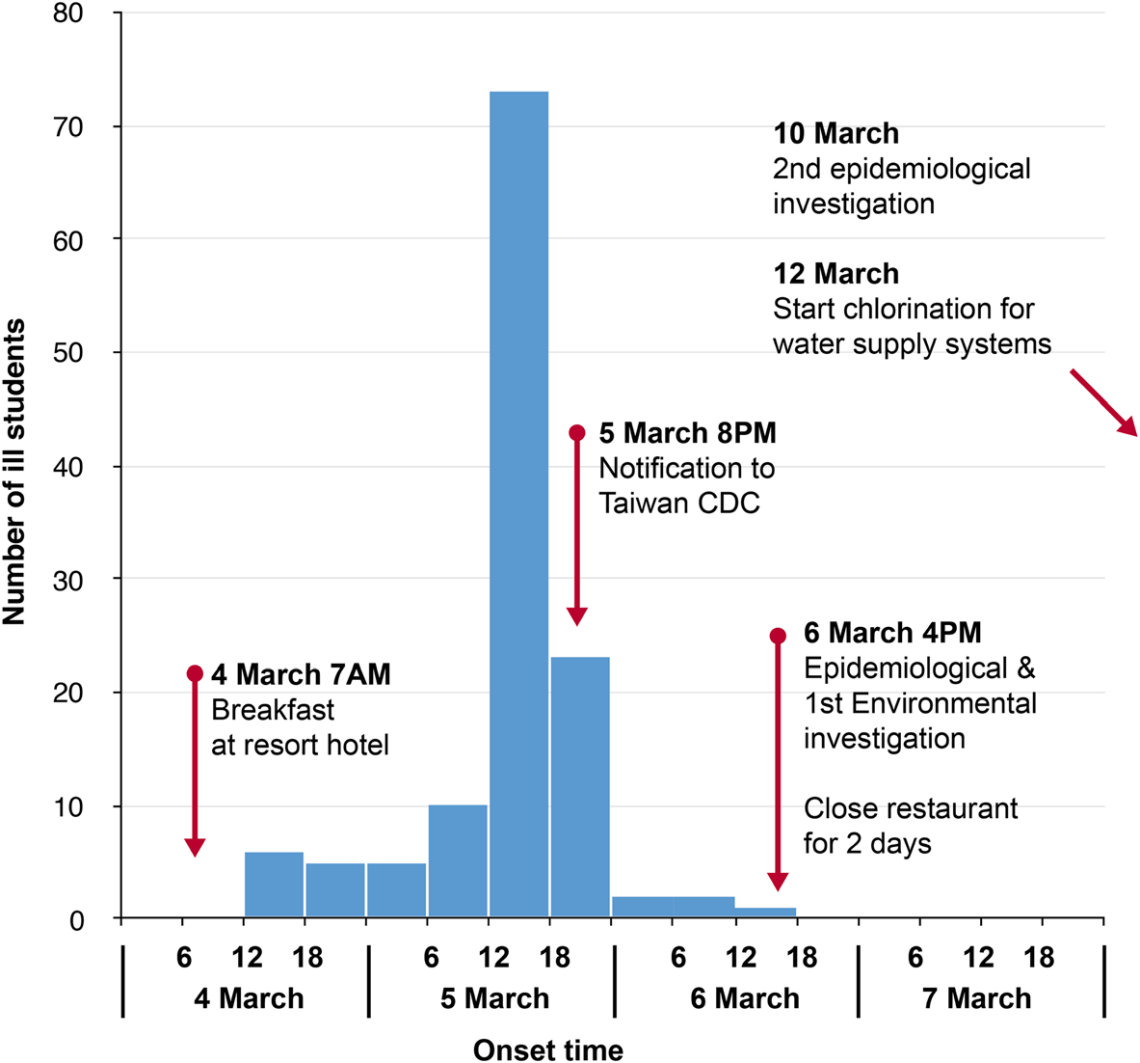
The epidemic curve of gastroenteritis cases in students. *N* = 127, because four students reported having illness on 5 March but did not mention the exact hour

A total of 275 (96%) of 285 students completed the survey. Eight students were excluded from analysis because of missing data or illness onset before the implicated breakfast on 4 March. Of 267 students included in the analysis, 144 (54%) met our case definition. Median age of cases was 17 years (range 16–26) and male-female ratio was 1.29. The epidemic curve showed a single wave pattern and median incubation period using the breakfast on 4 March as the start point was 32 h (range 4–47) (Fig. 1). Most common symptoms were nausea (78%), vomiting (69%), dizziness (64%), abdominal pain (60%), weakness (54%), fever (47%), and diarrhea (42%).

The following statistical analysis was focused on the breakfast on 4 March based on the onset time of students’ illness, estimated incubation period of norovirus and less than a half of students consumed 5 March breakfast because 22 students had already developed symptoms. In bivariate analysis, only those who consumed iced tea, including lemon tea or milk tea, had increased risk for gastroenteritis (risk ratio [RR] 1.54, 95% confidence interval [CI] 1.22–1.98) (Table 1). Regression analysis revealed similar findings in students who consumed iced tea (RR 1.48, 95% CI 1.17–1.84) (Table 2).

**Table 1:** Bivariate analysis of food items at 4 March 4 breakfast for gastroenteritis in students (*n* = 267)

Food items	Ate			Not ate			RR	*p value*
	Ill	Not Ill	AR	Ill	Not Ill	AR		
French Fries	88	78	53%	56	45	55%	0.96	0.70
Carbbage	22	24	48%	122	99	55%	0.87	0.39
Water spinach	7	12	37%	137	111	55%	0.67	0.19
Other vegetables^a^	6	8	43%	138	115	55%	0.79	0.44
Scramble eggs	64	54	54%	80	69	54%	1.01	0.93
Pasta	97	87	53%	47	36	57%	0.93	0.55
Tofu	9	10	47%	135	113	54%	0.87	0.58
Toast	61	49	55%	83	74	53%	1.05	0.68
Buns	30	32	48%	101	104	49%	1.06	0.64
Other breads^b^	1	8	11%	130	128	50%	0.61	0.30
Pineapple	1	4	20%	130	132	45%	1.11	0.77
Guava	9	9	50%	135	114	54%	0.92	0.74
Other fruits	2	3	40%	142	120	54%	0.74	0.58
Ham	23	11	68%	121	112	52%	1.26	0.07
Sausage	15	10	60%	129	113	53%	1.14	0.42
Orange juice	49	47	51%	95	76	56%	0.92	0.48
Drinking water	19	24	56%	120	104	54%	1.04	0.78
Iced lemon tea	34	18	73%	106	109	49%	1.48	<0.001*
Iced milk tea	54	33	62%	90	90	50%	1.24	0.05*
Iced lemon tea or milk tea	86	45	66%	58	78	43%	1.54	<0.001*

*AR* Attack Rate, *RR* Risk Ratio

^a^Other vegetables indicates those consumed vegetable other than cabbage and water spinach

^b^Other breads indicates those consumed breads other than Chinese steamed buns, round buns and toast.

*The *p* value is less than 0.05

**Table 2:** Multiple regression analysis of risk factors for gastroenteritis (*n* = 267)

Food items	Ate		Not Ate		RR*	95% CI
	Ill	Not ill	ill	Not ill		
Water spinach	7	12	137	111	0.75	0.42–1.33
Ham	23	11	121	112	1.20	0.93–1.53
Iced lemon tea or milk tea	86	45	58	78	1.48	1.17–1.87

*AR* Attack Rate, *RR* Risk Ratio

*Multiple logistic regression analysis was done by using log-binominal model and including the variables with *p* value <0.20 in bivariate analysis

### Environmental inspections

Staff of the restaurant, including 8 cooks and 10 waiters and waitresses, had regular health check-ups and their health status were all monitored. Only one staff had complained of symptoms of gastroenteritis but his symptoms resolved one week before this outbreak.

All cold dishes and cooked foods were prepared in the main kitchen by the same cooks. The iced tea was prepared by brewing tea powder with hot-boiled water and then adding ice into the bottles. Ice was made by ice machine using un-boiled but filtered tap water.

General hygiene condition in the restaurant met the requirements of Good Hygiene Practices. The resort was served by two water sources. Source A was tap water system that supplied to restaurant and its kitchen. Source B was a dual system, which was ground water mixed with tap water and supplied for daily lives in other parts of the resort. The ice machine used un-boiled tap water from Source A filtered with 5-micon pore size filter.

### Clinical and environmental specimens

Human specimens were collected from 10 waiters and waitresses, 8 cooks and 25 ill students. All stool and vomitus samples were negative upon bacterial examination; however, five vomitus and 11 stool samples from ill students tested positive for norovirus. The major genotype (13 of 16 students, 80%) was GII.17 (Table 3). One waiter tested positive for norovirus GI.4, but his symptoms had subsided one week before this outbreak and he denied any contact with foods or beverages consumed by students.

**Table 3:** Findings of norovirus genotypes in human patients and environmental samples in the outbreak, March 2015

Sample	Sample type	Onset time	Date of collection	GI genotype	GII genotype
Human					
Patient 1	stool	6 March 13:00	6 March		GII.17
Patient 2	stool	6 March 1:00	6 March		GII.17
Patient 3	stool	5 March 23:00	6 March		GII.17
Patient 4	stool	5 March 21:00	6 March		GII.17
Patient 5	stool	5 March 18:00	6 March	GI.2	
Patient 6	stool	5 March 18:00	6 March		GII.17
Patient 7	stool	5 March 18:00	6 March		GII.17
Patient 8	stool	5 March 18:00	6 March		GII.17
Patient 9	stool	5 March 18:00	6 March		GII.17
Patient 10	stool	5 March 14:00	6 March	GI.2	GII.17
Patient 11	stool	5 March	6 March	GI.4	GII.17
Patient 12	vomitus	5 March 18:00	6 March		GII.17
Patient 13	vomitus	5 March 18:00	6 March		GII.17
Patient 14	vomitus	5 March 18:00	6 March	GI.3	
Patient 15	vomitus	5 March 18:00	6 March	GI.4	
Patient 16	vomitus	5 March 18:00	6 March		GII.17
Environmental site					
Faucet in scullery	water		8 March		GII.17
Outlet of water pipe	water		8 March		GII.17
Before the filters of ice machine	water		8 March	GI.2, GI.4	GII.17
After the filters of ice machine	water		8 March	GI.2, GI.4	GII.17
B1 ice machine	ice		8 March	GI.2, GI.4	GII.17

**Fig. 2:**
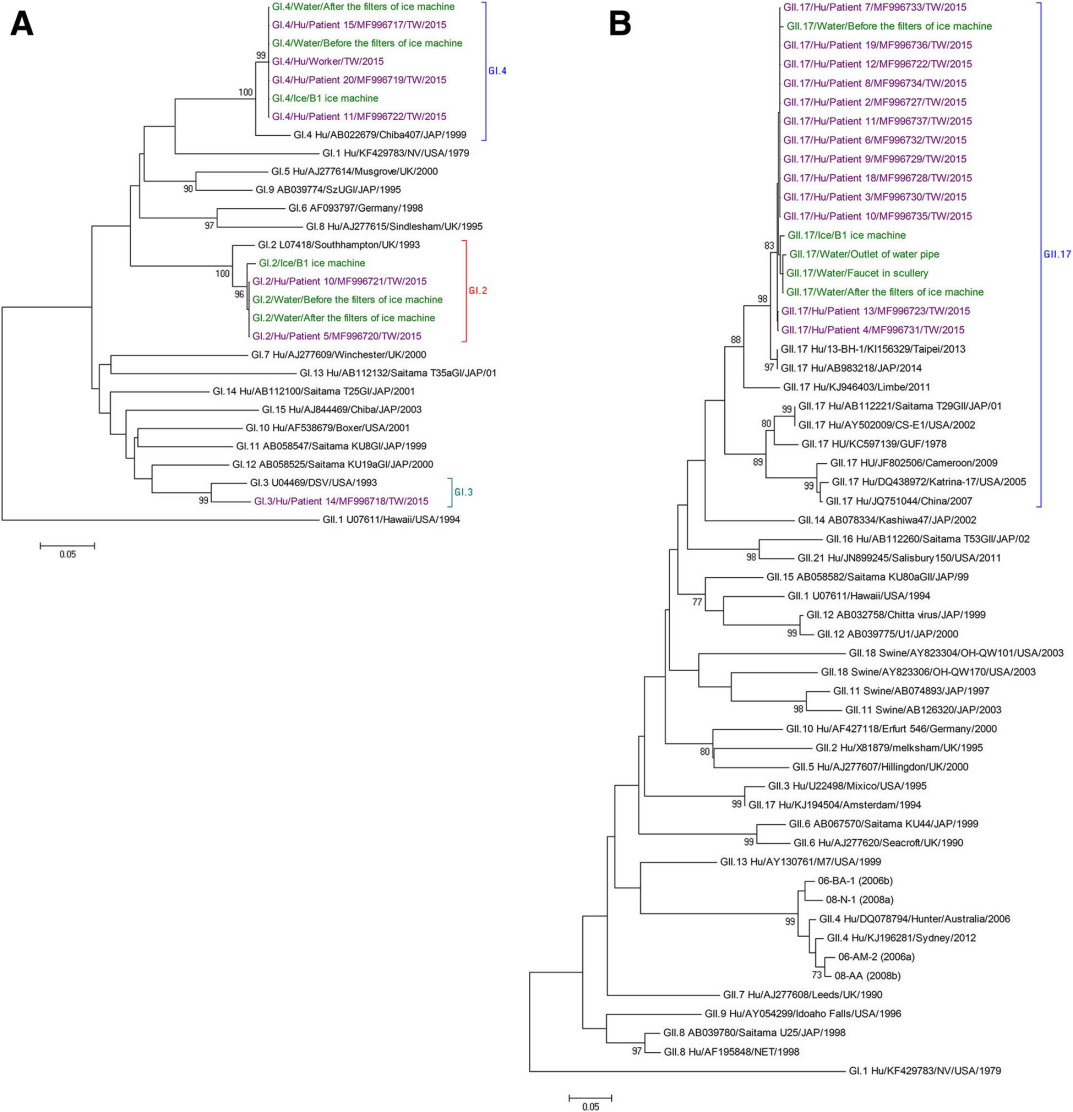
Phylogenic tree of partial noroviruses VP1 gene in human and water specimens. (**a**) genogroup I (**b**) genogroup II of partial norovirus capsid VP1 gene sequences (GI according to reference sequence KF306212 at 5339–5668; GII according to reference sequence KU561249 at 5051–5373) were aligned and phylogenetic trees were generated using the neighbor-joining method by MEGA 4.0 software. Bootstrap values of 1000 replications are shown on the branches. Green color and purple color indicate norovirus from the water and human specimens, respectively. Reference sequences from GenBank are named by genotype, accession number, country and year of detection. The GenBank accession numbers of the GII.17 sequences from patient 1~4, 6~13, 16~19 are MF996723-MF996738 and the GI strains sequences from patient 5, 10, 11, 14, 15 are MF996717- MF996722

No leftover of breakfast on 4 and 5 March were available for testing. Total 17 water samples taken from the kitchen, water source of ice machine and ice (from source A), ground water supply system of other departments (from source B) were tested. Two water samples collected from the faucet in the washing area in the first floor kitchen (from source A) and from the outlet of the water pipe of the ground water system (from source B) were positive for norovirus. In addition, the ice in the ice machine and the un-boiled water before and after the filters of ice machine were all tested positive for multiple variants of norovirus, including genotypes GI.2, GI.4 and GII.17. Further sequence and phylogenetic analyses of partial VP1 genes showed that 99–100% identified in the human and water specimens and clustered into same genotypes in GI.2, GI.4, and GII.17, respectively (Fig. 2a, b).

### Control measures

The restaurant was immediately closed for two days after the outbreak was reported. The pipelines of the water supply systems were chlorinated after the contamination of water system was confirmed. In order to check the clearance of the norovirus contamination in the water system, the specimens from the positive environmental site and water source were collected again for laboratory examination. Follow-up norovirus tests of water samples from the water supply systems and ice were all negative. No more cluster of gastroenteritis cases was noticed afterwards.

## Discussion

Based on epidemiologic, laboratory, and environmental evidence, this outbreak of acute gastroenteritis in Kenting was caused by norovirus, which was spread through waterborne transmission. Epidemiological analysis suggested the iced tea was the most likely vehicle for transmission. Sequence analysis showed both tap water and ground water were mainly contaminated by norovirus GII.17, which was clustered into the same genotypes with the human strain.

The epidemiological curve illustrated a point-source outbreak indicating possible foodborne or waterborne transmission. With application of molecular epidemiology in our investigation, we can confirm the source of the pathogen and provide solid laboratory evidence. Regarding both food-borne and water-borne norovirus outbreaks can be caused by multiple genotypes of norovirus [17] and the common existence of norovirus in the natural environment such as ground water [18, 19], it is often difficult to figure out whether norovirus found in the environment is the causative pathogen of illness in patients. By using phylogenic analysis, we confirmed that the norovirus found from ice and water belonged to the same clusters of genotypes as those found from human samples. This evidence combined with the findings of our epidemiologic investigation provided a stronger evidence for identification of the source and transmission route, which is important for health authorities to implement effective outbreak interventions.

Although the restaurant had a filtration system for the ice machines, our investigation discovered that it was not effective against norovirus because the filtration system had pore-size of 5 μm, and norovirus with same genotypes were found from ice and water before and after filtration. According to the recommendations of the US CDC [20], only nanoflitration or reverse osmosis has high effectiveness in removing norovirus. Adequate water treatment in these ways should be recommended to prevent waterborne outbreaks especially when ice is made from un-boiled water.

Ice-associated norovirus outbreaks have been reported several times because ice was usually made by un-boiled water and the risk of contamination was easily neglected [21, 22]. Current regulation for ice in Taiwan only requested that the source water must meet the drinking water quality standards. However, the microbiological indicators used in the standards, such as total bacterial count and coliform levels in water, have been proven non-predictive for norovirus [23]. On the other hand, routine viral tests for water sources are not feasible because of its high cost, although monitoring of norovirus has been proposed in the third Unregulated Contaminant Monitoring Rule in US [24]. Chlorination is generally considered effective for norovirus disinfection [25], but it is not obligatorily used for treatment of ground water in Taiwan. In dual public water systems using both treated tap and ground water, it might be safer to adapt the regulation of small water treatment facilities and to use chlorination for ground water because contamination can not be completely avoided.

The laboratory examination in our investigation showed the outbreak was caused by multiple variants of norovirus, including GI.3, GI4, and predominantly GII.17. This finding is not uncommon in waterborne norovirus outbreak, especially when strong sewage contamination was noticed [26]. Although norovirus GI was more predominant in previously reports of waterborne outbreaks, our investigation revealed the potential of GII.17 to cause large waterborne outbreak. We also find one waiter tested positive for only norovirus GI.4. However, his symptoms had subsided one week before this outbreak, suggesting his infectiousness was low and he denied any contacts with the students as well. Therefore, we considered he was another patient accidentally infected via contaminated water one week before rather than being the index case.

Previously, GII.4 was the most important genotype of norovirus because new variants caused cyclic epidemics every 2 to 4 years. Norovirus GII.17 was only sporadically reported in ground water before 2014. However, recent studies reported high frequency of GII.17 in ground water in Kenya [27] and in sewage or costal water in Asian countries including Korea, China and Japan [7, 18, 28]. Outbreaks caused by a new variant of GII.17 have also increased since late 2014 [18, 29, 30]. A survey conducted in Korea revealed GII.17 was the most prevalent strain of norovirus in asymptomatic food handlers [6] and the proportion of GII.17 detected in human norovirus cases was also increasing in China and Ethiopia [31, 32]. Our investigation suggests that this new variant GII.17 can cause large waterborne outbreaks. Public health professionals should maintain a high index of suspicion of water and ice as the source of large norovirus outbreaks because water-borne outbreaks usually result in larger size and higher attack rate than those spread through direct person-to-person contact. We recommend ongoing collaboration between human, food, and environmental health authorities during investigations and re-evaluating drinking water standards in response to the challenges raised by waterborne norovirus outbreaks.

There are some limitations in our investigation. First, recall bias is difficult to avoid since our investigation used a self-reported questionnaire. However, the response rate to the questionnaire was high and the elapsed time from the implicated breakfast to interviews was short. Thus, the recall bias could be minimized. Second, it is difficult to determine exactly where the contamination occurred in the ground water and the treated water system in the resort because we did not trace out pipelines used in the two systems. Thus, the local health authorities used chlorinating tablets in the whole water system to prevent further transmission of norovirus until no norovirus was detected. Last, not all students who consumed contaminated iced drinks developed gastroenteritis. The reason may be the cross-protection from prior norovirus exposure or other host factors but our investigation could not provide this information. We could not conclude the clinical characteristics and the tendency on age because this outbreak occurred in a close student tourists group and no other tourists visit the resort hotel restaurant at the same time as well. Further investigations may be worthwhile to delineate host factors that predispose these students to GII.17 infection and the clinical characteristics.

## Conclusion

By using molecular methods, we confirmed a norovirus gastroenteritis outbreak predominantly caused by GII.17. The source of infection was norovirus-contaminated ice made from un-boiled water. Adequately filtered or boiled water should be used to make ice, especially when dual treated and ground water supply system was established in the facilities.

## Abbreviations

CDC: Centers of Disease Control
ELISA: Enzyme-linked immunosorbent assay
FDA: Food and Drug Admisnistration
RT-PCR: Reverse transcription polymerase chain reaction
